# Beacon of Hope for Age-Related Retinopathy: Antioxidative Mechanisms and Pre-Clinical Trials of Quercetin Therapy

**DOI:** 10.3390/antiox14050561

**Published:** 2025-05-08

**Authors:** Ning Pu, Siyu Li, Hao Wu, Na Zhao, Kexin Wang, Dong Wei, Jiale Wang, Lulu Sha, Yameng Zhao, Ye Tao, Zongming Song

**Affiliations:** 1 Department of Ophthalmology, Henan Eye Hospital, Henan Provincial People’s Hospital, People’s Hospital of Zhengzhou University, Zhengzhou 450003, China; pun@stu.zzu.edu.cn (N.P.); q1w1e1a1s1d@stu.zzu.edu.cn (H.W.); wd5421@stu.zzu.edu.cn (D.W.); jlw@stu.zzu.edu.cn (J.W.); sha0309@stu.zzu.edu.cn (L.S.); zym200312@stu.zzu.edu.cn (Y.Z.); 2 College of Medicine, Zhengzhou University, Zhengzhou 450001, China; lsiyu@gs.zzu.edu.cn (S.L.); zn0114@gs.zzu.edu.cn (N.Z.); wkx20000121@gs.zzu.edu.cn (K.W.)

**Keywords:** oxidative stress, age-related retinopathy, quercetin, pharmacological effects

## Abstract

Age-related retinopathy is one of the leading causes of visual impairment and irreversible blindness, characterized by progressive neuronal and myelin loss. The damages caused by oxidation contributes to the hallmarks of aging and represents fundamental components in pathological pathways that are thought to drive multiple age-related retinopathies. Quercetin (Que), a natural polyphenol abundant in vegetables, herbs, and fruits, has been extensively studied for its long-term antioxidative effects mediated through diverse mechanisms. Additionally, Que and its derivatives exhibit a broad spectrum of pharmacological characteristics in the cellular responses of age-related retinopathy induced by oxidative stress, including anti-inflammatory, anti-neovascularization, regulatory, and neuroprotective effects in autophagy and apoptosis processes. This review mainly focuses on the antioxidative mechanisms and curative effects of Que treatment for various age-related retinopathies, such as retinitis pigmentosa, diabetic retinopathy, age-related macular degeneration, and glaucoma. Furthermore, we discuss emerging technologies and methods involving Que and its derivatives in the therapeutic strategies for age-related retinopathies, highlighting their promise for clinical translation.

## 1. Introduction

The retina is a photosensitive tissue located in the posterior segment of the eyeball that detects light stimuli and generates neural impulses [1]. Anatomically, the retina is considered a peripheral component of the central nervous system. In fact, similar to other parts of the brain, it is generally believed that once the retina deteriorates, its function cannot be regenerated or restored. Oxidative stress plays a crucial role in aging-related retinopathy [2]. External light reaches the retina through the refractive system of the eyeball. Both ultraviolet light from natural light and blue light from light-emitting diodes in electronic products can cause photo-oxidative stress [3,4]. Meanwhile, to generate the visual evoked signals, the retina needs to maintain high metabolic activity and withstand high oxidative pressure. Inevitably, a large quantity of reactive oxygen species (ROS) is produced within the mitochondria. ROS have highly reactive abilities to compete for the paired electrons of intracellular molecules and are continuously generated during the aging process. If the excessive ROS cannot be cleared in time, they will cause damage to chromosomal and mitochondrial DNA by modifying bases and inducing breaks in DNA strands [5,6,7,8]. The cytotoxic ROS can also oxidize sulfhydryl groups and modify amino groups, thereby disrupting protein molecular structures and affecting the function of signal transduction pathways [5]. The excessive accumulation of ROS eventually leads to mitochondrial dysfunction, triggers cellular apoptosis, promotes inflammatory responses, facilitates lipid peroxidation, and induces structural and functional impairments in the retina [9]. These retinal lesions further affect the visual function of patients and eventually lead to blindness. The damages caused by oxidation contribute to the hallmarks of aging and represent essential components in pathological pathways implicated in driving multiple age-related diseases [10]. Typically, age-related retinopathy mainly includes retinitis pigmentosa (RP), age-related macular degeneration (AMD), diabetic retinopathy (DR), leber congenital amaurosis, and hereditary retinal dystrophies, including stargardt disease [11,12]. To date, ophthalmic surgery, ophthalmic lasers, and ophthalmic drugs remain the three primary approaches for treating eye diseases, with pharmaceuticals continuing to play a crucial role in the diagnosis, treatment, and prevention of such conditions [13,14]. In clinical practice, surgical complications are difficult to avoid, including latrogenic retinal breaks, macular folds, cataract formation, recurrent subretinal hemorrhage, elevated intraocular pressure, and structural damage to the underlying retina [15,16]. During the process of pharmacological treatment, issues like poor bioavailability, toxicity, side effects, and high economic costs lead to the scarcity of truly clinically approved drugs, despite many types of drugs having been used for research. These age-related retinopathies lack efficient treatments due to their complex pathologic backgrounds and chronic processes.

Flavonoids constitute a large and structurally diverse class of polyphenolic compounds that are ubiquitously present in plant-derived foods. Plant polyphenols have been a subject of scientific interest for many decades. Flavonoids represent the most prevalent class of plant polyphenols and are primarily responsible for the flavor and color characteristics of fruits and vegetables. Among them, more than 6000 flavonoids have been identified [17]. Flavonoids have six major subclasses including the flavones (e.g., acacetin, apigenin, tangeretin, luteolin, and wogonin), flavonols (e.g., kaempferol, isorhamnetin, and myricetin), flavanones (e.g., hesperidin, pinostrobin, taxifolin, and naringenin), catechins or flavanols (e.g., epicatechin), isoflavones (e.g., genistein, glycitein, daidzein, formononetin, biochanin A, and irilone), and anthocyanidins (e.g., cyanidin and pelargonidin) [18,19,20,21,22,23]. Quercetin (Que) is a natural flavonol widely distributed in vegetables, herbs, and fruits. Its name is derived from the Latin word quercetum, which means oak forest [24,25]. Que contains multiple hydroxyl groups and four reactive groups: the dihydroxy group in the A-ring, an o-dihydroxy group in the B-ring, a C2–C3 double-bond in the C-ring, and a 4-carbonyl group [26]. Que exists in its various glycoside forms in numerous plant species, including hypericum ircinum, apium graveolens, lactuca sativa, prunus domestica, and cocoas, by attaching a glycosyl group (a sugar such as glucose or rutinose) as a replacement for one or more hydroxyl groups (commonly at position 3). Thus far, Que has exhibited a broad spectrum of biological activities, such as antioxidant, anti-fungal, anti-thalassemia, anti-obesity, anti-Alzheimer, anti-hypertension, cardio-protective, and anti-Parkinson’s activities, as well as anti-tumor activity [27]. Therapeutic trials have been conducted to evaluate the biological activity of Que in a series of diseases including tumors, allergic reactions, inflammation, arthritis, neurodegenerative brain disorders, cardiovascular diseases, liver and kidney diseases, lung diseases, etc. [28,29,30]. Encouragingly, with the widespread and in-depth research on Que, its therapeutic value in ocular diseases has also been explored and validated. Both experimental and clinical data have shown that dietary polyphenols supplements exert beneficial effects on visual function. In particular, its antioxidant and anti-inflammatory properties, along with other activities, may have an impact on the treatment of various ophthalmological conditions, including keratoconus, proliferative vitreoretinopathy, cataracts, Graves’ ophthalmopathy, conjunctivitis, retinopathy, and other ophthalmological diseases [31]. Thus far, Que has been widely used in the treatment of age-related retinopathy. In this review, we have described the pharmacological effects of Que treatment on various age-related retinopathies, including the RP, AMD, DR, and glaucoma.

Despite its numerous pharmacological advantages and long history of usage as a nutraceutical, the use of Que as a therapeutic molecule in clinical research remains limited. Researchers have attempted to design and synthesize many new Que derivatives to overcome the pharmacodynamic limitations, encompassing modifying the biological and structural activity relationships of Que derivatives on the original basis [32]. Additionally, nanotechnology represents a promising avenue of research with the potential to enhance the therapeutic efficacy of a range of compounds. The coupling of therapy with an efficient nanocarrier-based drug delivery system is essential to ensure bioavailability and selective targeting, as well as controlled Que release with minimized dosing frequency [33]. In this study, firstly, we describe the pharmacokinetics and pharmacological characteristics of Que. Secondly, we summarize the curative effects and molecular mechanisms of Que in the therapeutic strategies for age-related retinopathy. Thirdly, we comprehensively review new technologies and methods for treating age-related retinopathy based on nanostructures and analyze the strengths and weaknesses of the methods used in different studies. It is envisioned that continued refinement of this knowledge will provide novel horizons for the clinical application of Que in ophthalmological practice.

## 2. Pharmacokinetics of Quercetin

Que (C_15_H_10_O_7_) is a brilliant citron-yellow needle crystal. It is completely insoluble in both cold water and in hot water, but exhibits good solubility in alcohol and lipids [25]. Flavonols in their free forms, namely aglycones, have lipophilic properties. Due to its lipophilicity, Que can effectively penetrate the blood–retinal barrier following oral or systemic administration. These characteristics constitute the basis of how Que is used to treat degenerative retinopathies. Most of the Que forms synthesized by plants are attached to sugar conjugates as the glycosidic forms [32]. The hydroxyl functional groups on all three rings serve as sites for linkage to saccharide groups (i.e., O-glycosides) [33]. The saccharide groups attached to flavonols include the monosaccharides glucose, galactose, and arabinose [34]. Typically, dietary Que glycosides comprise Que-3-O-rutinoside(rutin), Que-3-O-glucoside(isoquercetin), and Que-3,4′-O-diglucoside [32]. Que is ingested in the form of glycosides by humans from plants, and their glycosyl groups are hydrolyzed during chewing, digestion, and absorption. Subsequently, Que glycosides are converted into aglycone in the intestine through the enzymatic action of β-glycosidases prior to being absorbed into enterocytes. Following this, the aglycones undergo phase I metabolism (oxidation and O-demethylation) and phase II metabolism (glucuronidation, methylation, and sulfation) within the enterocytes, resulting in the formation of metabolites [34,35]. However, due to the presence of enzymes in the gut that interact with Que, different chemically modified Que forms may have different yields on the extent of absorption. In the liver, the remaining aglycones are metabolized into methyl, glucuronide, and sulfur metabolites [35,36,37]. In humans, plasma Que primarily exists in the conjugated forms and is subsequently distributed throughout the entire body’s tissues via the bloodstream [38]. It is reported that the half-life of Que ranges from 11 to 28 h, with final excretion occurring via the urinary system [39]. However, during their residence in the bloodstream, metabolic modification alters the nature of Que xenobiotic compounds. For instance, the glucuronide and sulphate conjugates are the predominant forms of Que in plasma [40]. The quercetin-3-O-β-D-glucuronide retains the catechol group, exhibiting enhanced stability, and thus more effectively prevents oxidative stress than Que aglycone [41]. In contrast, tamarixetin and isorhamnetin are O-methylated in the B-ring, which results in reduced antioxidative activity. However, tamarixetin has the highest anti-inflammatory activity among Que and its metabolites [42]. Even when Que is taken orally, the human intestinal microbiota can metabolize Que, and there are differences in microbial Que metabolism between individuals [43]. It can be postulated that the potential of Que to mitigate retinal damage may partly rely on its metabolites. However, it is regrettable that the accumulation of these metabolites in the retina has yet to be verified. Further clinical trials are in emergent need to unravel the metabolic mechanisms of Que and its derivatives in the human eye.

The content of Que in the diet is typically low, with daily flavonol intake estimated at approximately 16 mg/day [44]. The normal intake of Que appears to play a limited regulatory role. However, due to its reported beneficial effects on health, Que supplements are available with recommended doses of up to 2 g/day [44]. When used as a pharmaceutical agent, the maximum recommended adult daily dose of Que can reach up to 4000 mg [45]. Nevertheless, the potential effects of such high doses on humans require further assessment. Additionally, different administration methods can influence the absorption percentage of Que, thereby affecting both the therapeutic efficacy and pharmacokinetic profiles. One research indicates that a single oral dose of 10 mg/kg Que given to rats via gavage resulted in only 5.3% for unchanged Que within 24 h, despite the total absorbed Que reaching 59.1% [46]. In this process, approximately 93.3% of the oral Que dose is metabolized in the intestine into other forms for absorption, primarily its glucuronide and sulfate conjugates. Additionally, several studies have demonstrated that glycosylated forms of Que are more readily absorbed compared with unchanged Que. For example, Hollman et al. [47] investigated Que absorption in nine ileostomy patients. After a 12-day Que-free diet, participants were randomly assigned to one of two diets for 12 days: fried onions (containing Que glucosides) or 100 mg of pure Que aglycone. Results showed that the oral absorption of Que aglycone was approximately 24%, while the absorption of Que glycosides from onions reached 52%, indicating that the glycoside structure enhances absorption. However, intraperitoneal administration of Que results in higher tissue concentrations, such as in tumors, compared with oral intake [48]. Local administration of Que directly into the ocular tissue, such as via intravitreal injection, can reduce metabolism and first-pass elimination before Que reaches the eye. However, a comprehensive comparative analysis across all administration routes would require additional controlled experiments.

## 3. Pharmacological Characteristics of Quercetin in the Cellular Responses of Age-Related Retinopathy Induced by Oxidative Stress

**Figure 1:**
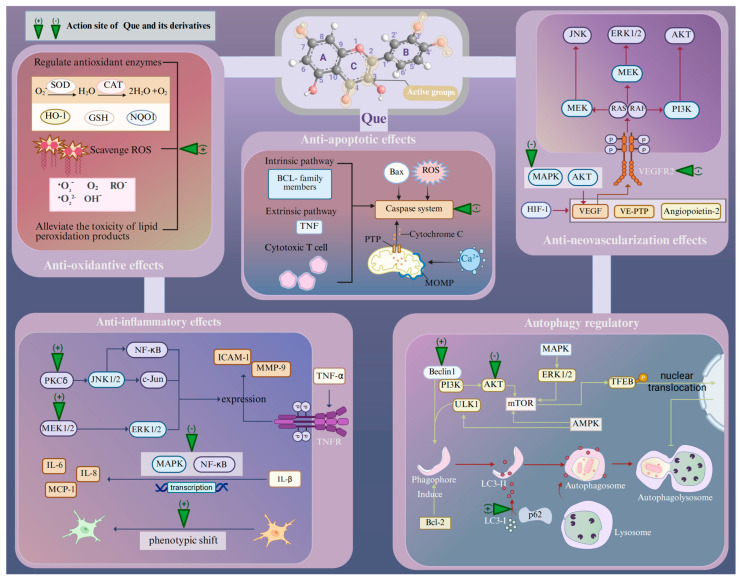
Pharmacological characteristics of Que in the treatment of age-related retinopathy. Abbreviations: Que, quercetin; SOD, super oxide dismutase; CAT, catalase; GSH, glutathione; HO-1, heme oxygenase-1; NQO1, NAD(P)H quinone oxidoreductase-1; TNF-α, tumor necrosis factor α; IL, interleukin; MMP-9, matrix metalloprotease-9; ICAM-1, intercellular adhesion molecule-1; PCKδ, protein kinase C delta; MEK1/2, mitogen-activated extracellular signal-regulated kinase 1/2; JNK1/2, c-Jun N-terminal kinase 1/2; NF-κB, nuclear factor kappa-B; ERK1/2, extracellular signal-regulated kinase 1/2; MAPK, mitogen-activated protein kinase; AKT, protein kinase B; mTOR, mammalian target of rapamycin; HIF-1, hypoxia inducible factor-1; VEGF, vascular endothelial growth factor; VE-PTP, vascular endothelial protein tyrosine phosphatase; Bcl-2, B-cell lymphoma 2; Bax, Bcl-2-associated X protein; ROS, reactive oxygen species; PTP, permeability transition pore; MOMP, mitochondrial outer membrane permeabilization; LC3, light chain 3; PI3K, phosphatidylinositide 3-kinase; AMPK, AMP-activated protein kinase; p62, prostacyclin; TFEB, transcription factor EB nuclear. Created with BioRender.com.

Age-related retinopathies have different etiologies and pathogeneses, but at the molecular and cellular levels, their responses to retinal injury are similar [49]. The presence of multiple oxidative stress mechanisms within the retina suggests that the ROS-mediated protein and lipid modifications may have a detrimental effect on signal transduction pathways [9]. For example, ROS overproduction by chronic oxidative stress can lead to the modification and damage of protein folding, membrane lipids, nucleic acids, enzymatic activities, and cell-surface conditions, including the receptor expression and plasma membrane potential, as well as the cytoskeleton that provides support for various organelles [50]. These effects, originally a compensatory system, may finally result in pathological events including inflammation, disorganized autophagy, endoplasmic reticulum (ER) stress, and retinal neovascularization (RNV) [50]. Then, apoptosis, necrosis, and ferroptosis act as major mechanisms for retina death in response to oxidative stress [50,51]. Que can protect against neuronal damage in the retina by inhibiting oxidative stress and the cellular responses associated with age-related retinopathy induced by oxidative stress (Figure 1).

### 3.1. Oxidative Stress

As a natural flavonoid, Que can provide long-term antioxidative effects through diverse mechanisms. Specifically, Que contains four active groups: a dihydroxy group between the A-ring, O-dihydroxy group B, C-ring C2–C3 double bond, and 4-carbonyl [52]. The key to Que’s high antioxidant capacity is the number and relative position of its hydroxyl groups, as well as the size of the condensed π-system [53]. As hydroxyl groups are replaced, the antioxidant capacity of Que derivatives decreases, but Que derivatives with the same number of hydroxyl groups exhibit varying antioxidant capabilities due to differences in the relative positions of their hydroxyl groups. The optimum stimulating effect is observed when the hydroxyl groups attached to Que are separated by an even number of carbon atoms [54]. Moreover, the hydroxyl groups in Que can also synergistically scavenge radicals, and the efficiency of this cooperation action depends on their relative positions. Specifically, adjacent hydroxyl groups can increase the potential antioxidant activity by stabilizing the flavone radical through intramolecular hydrogen-bonding interaction [53,55]. Additionally, 3-hydroxyl group is the active center in the AC-ring and its activity is positively influenced by the hydroxyl groups located at the 5 and 7 positions within the AC-ring [53]. Several lines of evidence suggest that Que can scavenge various free radicals, such as superoxide anion, peroxyl, alkoxyl, quench singlet oxygen, and hydroxyl radicals [38]. Moreover, ROS can cause lipid peroxidation, which further damages cell membranes, resulting in increased membrane permeability and the overproduction of harmful intermediates. Que is also able to alleviate the toxicity of lipid peroxidation products in AMD. For instance, 4-hydroxynonenal (HNE) is a highly cytotoxic aldehyde formed during the process of lipid peroxidation [56]. Furthermore, Que demonstrates the ability to regulate the expression levels of antioxidant enzymes. In streptozotocin-induced diabetic rats, retinal glutathione (GSH) levels were significantly lower (approximately 4 nmoles/mg protein) compared with normal rats (approximately 16 nmoles/mg protein). However, in Que-treated rats (50 mg/kg body weight, orally for six months), retinal GSH levels were significantly higher (approximately 12 nmoles/mg protein) than in the diabetic group [57]. There is also a positive modulation of antioxidant enzyme activities, particularly superoxide dismutase (SOD) and catalase (CAT) [57].

The antioxidant capacity of Que derivatives is modulated by different chemical groups. Correspondingly, adding methyl groups to Que can enhance its antioxidant characteristic. This enhancement can be attributed to the radical-scavenging reaction, wherein Que transfers electrons to radicals, forming a Que radical cation intermediate. The presence of electron-donating units, such as methyl groups ortho to the hydroxyl units of the catechol in the structure of Que, increases its radical scavenging activity [27]. For instance, C-8-aminomethyl analogs of Que exhibit a greater protective effect on erythrocytes against acute oxidative stress induced by H_2_O_2_ [27]. Notably, its performance of antioxidant activity is influenced by the spatial structure of Que, such as the radical cation of compound methyl Que that is stabilized through its planar structure between the 4H-curomen [27,58]. Consequently, it is evident that Que and its derivatives exert antioxidant effects through multiple mechanisms.

### 3.2. Inflammation

Inflammation protects an organism from harmful substances and pathogens, while its overactivation also causes detriment to the organism. Age-related retinal diseases are often caused by the disruption of retinal homeostasis and the presence of low-grade chronicin flammation (para-inflammation) [59]. It is acknowledged that the positive feedback loop between ROS and inflammation plays a pivotal role in the pathogenesis of the age-related retinopathy [60]. Chronic inflammation exacerbates the retinal damage over time and ultimately leads to the occurrence of age-related retinopathy [59,61]. It has been reported that inflammatory cells such as microglia and infiltrating macrophages play a persistent role in the development of age-related retinopathy. Additionally, the pro-inflammatory response of microglia, along with the activation of the complement system towards damaged neurons and debris, exacerbates immunopathology [59,61]. For instance, Toll-like receptors, retinoic acid-inducible gene I-like receptors, and nucleotide oligomerization domain (NOD)-like receptors (NLRs) can activate the inflammatory reaction when the retina is exposed to noxious stimuli [62]. The inflammatory cascade leads to the formation of NLR thermal protein domain-associated protein 3 (NLRP3) inflammasome in microglia, which subsequently up-regulates the expression of caspase-1 [63]. Moreover, physical barriers, including the inner and outer blood–retinal barrier, seem to be compromised as a result of the age-related dysfunction of endothelial and glial cells within the neurovascular unit and retinal pigment epithelium (RPE) cells [64]. Further exploration of ways to suppress inflammation could provide new insights into the treatment of age-related retinopathy.

Que has exhibited potent anti-inflammatory effects in multiple types of organisms. The anti-inflammatory characteristics are in line with those of flavonoids, which have a flat cyclic structure with C2-C3 unsaturated bonds and hydroxyl groups in specific positions. Que has been shown to lose its anti-inflammatory activity when the hydroxyl groups in the 3′ and 4′ positions of the B-ring are missing [65]. Que attenuates the inflammatory response of human RPE cells (ARPE-19 cells) against H_2_O_2_-induced oxidative injury. This is achieved by inhibiting the transcription of inflammatory factors such as cyclooxygenase-2 (COX-2), tumor necrosis factor α (TNF-α), and inducible nitric oxide synthase and decreasing the levels of nitric oxide (NO) in the culture medium [66]. Further research has indicated that Que may attenuate TNF-α-induced matrix metalloprotease-9 (MMP-9) and intercellular adhesion molecule-1 (ICAM-1) expression in ARPE-19 cells via the MEK1/2/ERK1/2 and PKCδ/JNK1/2/c-Jun or nuclear factor kappa-B (NF-κB) pathways in ARPE-19 cells [67]. Que has also been demonstrated to protect ARPE-19 cells from IL-1β-stimulated increases in IL-6, IL-8, ICAM-1, and monocyte chemoattractant protein-1 (MCP-1) production [68]. This is achieved by blocking the activation of the mitogen-activated protein kinase (MAPK) and NF-κB signaling pathways, thereby ameliorating the inflammatory response. The involvement of microglia is inevitable in the retinal inflammatory process of AMD and glaucoma progression [69]. The activation of microglia is essential to repair the injured tissue, as they can induce a robust response of the innate immune system and trigger the activation of adaptive immunity. The M1 phenotype of microglia can express inflammatory mediators (IL-1β, IL-6, and TNF-α) that are harmful to retinal photoreceptors and the blood–retinal barrier, triggering an inflammatory cascade. Conversely, the M2 phenotype induces the generation of anti-inflammatory cytokines (IL-1 receptor antagonist, IL-4, and IL-10) and promotes tissue repair [70]. In a retinal inflammation mouse model established by the intraperitoneal administration of lipopolysaccharide, pre-treatment of Que via intraperitoneal injection can induce a phenotypic shift in microglia from the M1 phenotype to the M2 phenotype, thereby alleviating retinal inflammation and stabilizing the integrity of the blood–retina barrier [70,71]. Further investigations are warranted to elucidate the precise mechanisms underlying the Que-mediated anti-inflammatory effects.

### 3.3. Retinal Neovascularization

RNV is commonly induced by hypoxia of ischemic retinopathy, which is an intractable pathological hallmark in many ocular blinding diseases. Chronic oxidative stress and inflammatory and protein aggregation processes may induce damage that contributes to choroidal neovascularization and subretinal fibrosis [72]. These newly formed retinal blood vessels are not robust vessels and they cannot undertake the arduous task of compensating for retinal ischemia. Conversely, RNV will cause deleterious complications, such as retinal hemorrhage and even retinal detachment [71]. Consequently, inhibiting the progression and development of RNV is critical in managing ischemic age-related retinopathies like DR.

Although the mechanism of its onset is exclusive, some common studies indicate that the production of hypoxia inducible factor-1 (HIF-1) plays a central role in the pathogenesis of RNV. HIF-1 induces an up-regulation in the expressions of hypoxia-regulated gene products, such as vascular endothelial growth factor (VEGF), vascular endothelial protein tyrosine phosphatase (VE-PTP), and angiopoietin-2. Collectively, these biological factors promote the generation of RNV [73]. Both clinical and experimental evidence suggests that VEGF facilitates the growth of RNV [74,75,76]. Several in vitro experiments have demonstrated that Que alleviates RNV by suppressing the invasion, migration, and tubular formation of microvascular dermal endothelial cells. Furthermore, Que inhibits VEGF-induced angiogenesis in the choroid and retina within the rhesus macaque choroid–retinal endothelial (RF/6A) cell line by specifically targeting the VEGFR-2 pathway [77]. Que can suppress VEGF-induced excessive inflammatory response in retinal photoreceptor cells through the inhibition of MAPK and protein kinase B (AKT), thereby inactivating NF-κB signals. In addition, the degradation of tight junction proteins in 661W cells (Zona occludins-1 and β-catenin) decreased by VEGF is recovered by Que [78]. The compound 8MQPM, a permethylated form of Que, is a potent inhibitor of angiogenesis both in vitro and ex vivo [79]. The compound 8MQPM can cause recoveries or the complete re-establishment of the transendothelial electrical resistance to the control values in human microvascular endothelial cells after stimulation with VEGF-A. Furthermore, it has been shown to suppress the activation of VEGFR2 downstream signaling molecules, including AKT, c-Jun N-terminal kinase (JNK), and extracellular signal-regulated kinase (ERK). These inhibit cell viability and migration and disrupt the formation of microvessels in the rabbit aortic ring. Therefore, the anti-neovascularization effects of Que may hold promise as a novel therapeutic agent for vascular-related retinopathy.

### 3.4. Apoptosis

Apoptosis is essential for normal cell turnover and tissue homeostasis and its mis-regulation is increasingly implicated in aging and aging-related disease [80]. Apoptosis is often the end fate of retinal cells responding to oxidative stress [1]. The chronic and progressive loss of neurons is a pathological marker of retinal degeneration [81]. The apoptosis of retinal neurons should be ascribed to different defects, such as overactivation of glia, disrupted synaptic connectivity, faults in axonal transport, neurotrophic factor deprivation, and the excessive release of neurotransmitters, as well as oxidative stress [82,83]. Failure to regulate apoptosis can lead to the pathological changes, as exhibited in age-related retinopathy. Since mammalian retinal neurons have a poor regenerative capacity, it is feasible to use Que to inhibit the photoreceptor apoptosis in age-related retinopathy.

Apoptosis is a genetically controlled programmed form of cell death. It is characterized by subcellular changes, such as the fragmentation of DNA, organelle swelling, and the formation of apoptotic bodies [84,85]. Apoptosis can be initiated by the intracellular sensors, extracellular immune factors, or biological signals from damaged cells as a means of removing unwanted cells. Typically, there are three pathways that can mediate apoptosis, including the intrinsic pathway dominated by B-cell lymphoma 2 (BCL-2) family members, the extrinsic pathway mediated by the TNF superfamily, and the third pathway mediated by cytotoxic T cells [86]. The initiation of apoptosis is dependent on a series of cysteine-aspartic proteases known as caspases, which act as the central proteases that all apoptotic pathways converge on [86]. In the mitochondrial-mediated pathway, the opening of the mitochondrial permeability transition pore (PTP), regulated by matrix mitochondrial outer membrane permeabilization (MOMP) and the pH of the mitochondria, causes the release of cytochrome c into the cytoplasm, thus resulting in the activation of the caspases [87,88,89]. The caspase-dependent cascade serves as a critical component in the initiation, transduction, and amplification of intracellular apoptotic signaling pathways [90]. The activation and function of the caspase system are regulated by various molecules, including inhibitors of apoptosis proteins, calpain, BCL-2 family proteins, and Ca^2+^ [90]. Previously, Que has been used to treat diseases through modulating the apoptotic pathways. Several lines of evidence suggest that Que exerts anti-apoptotic effects to protect against the loss of some cell types that are difficult to regenerate. Que has been demonstrated to attenuate the inflammatory response of ARPE-19 cells from H_2_O_2_-induced oxidative injury. This has been accompanied by an enhancement in BCL-2 transcript levels, an increase in the BCL-2/Bax ratio, and a suppression of the transcription of proapoptotic factors such as Bax, caspase-3 and caspase-9 [67]. In greater detail, Que down-regulates the protein expressions of Bax, cleaved caspase-3, and cleaved PARP and up-regulates the expression of BCL-2 through inhibiting the PI3K/AKT signaling pathway, which protects RPE and the retina from sodium iodate (NaIO_3_)-induced apoptosis [91]. An in vitro experiment showed that the administration of Que can reduce the thinning of all retinal layers induced by ischemia–reperfusion injury in the rat retina [92]. Moreover, Que not only improved the survival and function of retinal ganglion cells (RGCs) at a very early stage of chronic ocular hypertension in vivo, but also promoted the survival of hypoxia-treated primary cultured RGCs in vitro by ameliorating mitochondrial function and inhibiting mitochondria-mediated apoptosis [93].

### 3.5. Autophagy

Autophagy is a fundamental homeostatic pathway that mediates the degradation and recycling of intracellular components. According to the mechanisms by which substances are transported into lysosomes, autophagy can be divided into macroautophagy, chaperone-mediated autophagy, and endosomal microautophagy [94]. Among them, the most common type is macroautophagy, a process that begins with the formation of autophagosomes. The autophagosomes subsequently fuse with lysosomes to degrade the encapsulated cargo. The basic process of macroautophagy is regulated by many cytokines, including autophagy-related protein, microtubule-associated protein light chain 3 (LC3), Beclin 1, and sequestosome 1 (p62) [95].

Related clinical trials support that the efficiency of autophagy progressively declines as aging proceeds [96]. The inability to effectively remove aggregates may substantially contribute to the onset of age-related pathologies [96]. In the normal physiological process of the retina, autophagy is responsible for modulating vision quality, eliminating toxic aggregates, and promoting the recirculation of visual pigments [97]. In the retina, impaired autophagy is associated with lipofuscin accumulation, metabolic disruption, and increased oxidative stress [98]. In this context, an abnormal autophagy process will directly affect the structural morphology and physiological function of the retina, leading to the exacerbation of retinal diseases. Encouragingly, by activating autophagy, Que can degrade harmful cellular components such as misfolded proteins and peroxides, promote cell metabolism, and alleviate pathological changes. Que can directly initiate the autophagy process through inducing the conversion of LC3-I to LC3-II [99]. LC3-II is a typical biomarker of autophagic flux that can attach to the membrane of autophagosome and mediate the fusion of lysosomes with autophagosomes to form autolysosomes [100]. Moreover, Que promotes the consumption level of p62, a critical autophagy substrate, and activates Beclin 1, thereby promoting the formation of autophagic vacuoles and autophagosomes [101,102]. The mammalian target of rapamycin (mTOR) is a prominent regulatory molecule of autophagy. Activation of the mTOR pathway, mediated by AKT and MAPK signaling, inhibits autophagy, whereas the negative regulation of mTOR through AMPK signaling promotes autophagy. Que is a direct inhibitor of mTOR and can also promote transcription factor EB (TFEB) nuclear translocation, which is a downstream protein of mTOR, thereby inducing autophagy and lysosomal biogenesis in cultured RPE cells [103]. In particular, Que-mediated effects on autophagy also affect other biological processes within the cell. Que stimulates autophagy to reduce inflammation and total ROS accumulation, reducing oxidative damage and thereby bolstering the antioxidant defense system. However, the Que-mediated effect on autophagy is not always positive. Emerging evidence suggests that Que can inhibit autophagy. However, this has not yet been demonstrated in the retina. However, although it is often proposed that the decreased autophagy is detrimental and increased autophagy is beneficial, recent advances reveal that there exists a multifactorial relationship between autophagy and aging. The right balance of autophagy is highly specified for each stage of life and will likely benefit long-term health [96]. For example, a mild enhancement in autophagy has been shown to extend lifespan in flies, whereas an excessive increase in autophagy tends to shorten lifespan [104]. Therefore, more attention should be paid to the dual regulatory effect of Que on autophagy when using it as a therapeutic drug for age-related retinopathy.

## 4. Quercetin-Mediated Therapeutic Mechanisms on Age-Related Retinopathy

### 4.1. Age-Related Macular Degeneration

AMD is a serious age-related retinopathy that leads to severe visual impairments in the elderly population [105]. It is characterized by the progressive death of photoreceptors, the accumulation of lipofuscin and drusen, changes in the composition of the Bruch’s membrane, and chronic vascular inflammation [106]. Although multiple etiologic factors such as environment stimuli, genetic mutations, and lifestyle habits can lead to the onset of AMD, age itself remains the most important risk factor, making it an urgent priority to connect the underlying aging mechanisms with the pathophysiology of AMD [107]. In Western countries, one in every seven individuals over the age of 70- and one in three over the age of 80- is at risk of developing AMD [108]. However, as life expectancy escalates and global human societies undergo concomitant demographic shifts, AMD exacts a profound toll on the physical and mental health of the elderly and their families.

The pathological mechanisms operating in AMD progression encompass mitochondrial damage, elevated oxidative stress, dysregulated microglia and macrophage activities, and chronic inflammatory reactions [109]. A recognized early event of AMD is altered mitochondria in RPE cells, which subsequently induces oxidative damage [110]. ROS can destroy the structure of membrane proteins and DNA, stimulate excessive immune responses, and disrupt the function of RPE cells. The RPE malfunction eventually leads to the accumulation of lysosomal lipofuscin and extracellular drusen deposits. Lipofuscin is composed of autofluorescent vitamin A metabolites (bisretinoids). Bisretinoids are formed by the non-enzymatic condensation reactions of retinaldehyde and phosphatidylethanolamine within the discs of photoreceptors’ outer segments [109]. When the RPE cells engulf the outer segment discs, bisretinoids are trapped in the lysosomes due to their pH-dependent protonation [111]. They compromise the function of RPE by interfering with multiple homeostatic mechanisms. The earliest identified bisretinoid structure is N-retinylidene-N-retinylethanolamine (A2E) [112]. A2E and its oxidized forms, A2E-epoxides, can damage DNA in RPE cells [113]. A2E can photodecompose into glyoxal and methylglyoxal, which can trigger VEGF [109]. As the RPE cytoskeleton is altered, the accumulation of debris and drusen deposition between the RPE and the choroid is defined as dry AMD [114]. In the subsequent later stage, approximately 15% of dry AMD cases will evolve into wet AMD due to the altered balance of pro-angiogenic and anti-angiogenic factors [110].

**Figure 2:**
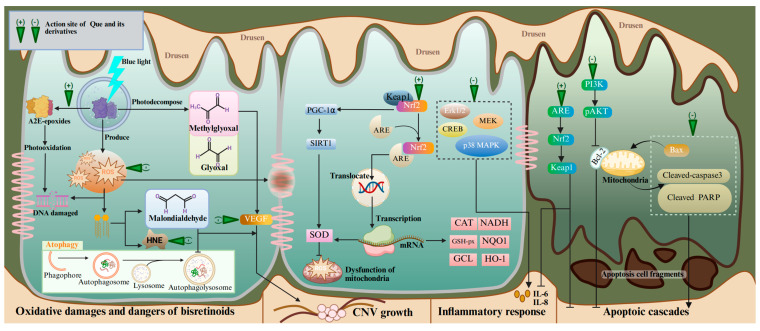
The molecular mechanisms and action sites of Que in the treatment of AMD. Abbreviations: A2E, N-retinylidene-N-retinylethanolamine; HNE, 4-hydroxynonenal; PGC-1α, peroxisome proliferator-activated receptor γ coactivator 1-alpha; SIRT1, silent information regulator 1; NRF2, nuclear factor erythroid 2-related factor 2; ARE, antioxidative response element; CREB, cAMP-response element binding protein; p38 MAPK, protein 38 mitogen-activated protein kinase; MEK, mitogen-activated extracellular signal-regulated kinase; NADH, nicotinamide adenine dinucleotide; GSH-px, glutathione peroxidase NQO1, GCL, glutamate cysteine ligase; CNV, choroidal neovascularization; KEAP1, Kelch-like ECH-associated protein 1; Cleaved PARP, cleaved poly ADP-ribose polymerase. Created with BioRender.com.

Emerging research has demonstrated the therapeutic value of Que in treating AMD on the basis of its antioxidant, anti-apoptotic, anti-angiogenic, and anti-inflammatory capacities (Table 1). Que has received the most research attention on antioxidant therapy for AMD, which can be divided into three main aspects: clearing ROS, regulating nuclear factor erythroid 2-related factor 2 (NRF2) and secondary enzymes, and inhibiting phototoxicity (Figure 2). Que can inhibit the generation of mitochondrial ROS by enhancing the deacetylated SOD2 levels in an AMD mouse model and ARPE-19 cells through the NRF2/PGC-1α/SIRT1 signaling pathway [115]. Under normal conditions, NRF2 binds to Kelch-like ECH-associated protein 1 (KEAP1) in the cytoplasm with a resting state. When the retina is exposed to oxidative stress, NRF2 dissociates from KEAP1 and translocates to the nucleus through combining with antioxidative response element (ARE). These biological activities lead to the up-regulated expressions of genes encoding the phase II metabolizing enzymes, antioxidase, and other stress-response mediators, including heme oxygenase-1 (HO-1), NAD(P)H quinone oxidoreductase-1 (NQO1), NADH, GSH-px, GCL, epoxide hydrolase, SOD, CAT, etc. [116,117,118]. An in vivo experiment using NRF2 knock-out rats further validates the regulatory effect of Que on NRF2 [119]. Excessive light exposure has been clearly recognized as a risk factor of phototoxicity at the early stages of AMD [120]. Phototoxicity leads to the formation of A2E-oxane, the accumulation of oxidative modified lipids such as malondialdehyde and 4-oxonanal, and the up-regulation of oxidative stress genes. Que can inhibit A2E photooxidation and alleviate the 4-oxonanal-mediated cytotoxic effects on the ARPE cell line through the ERK and MAPK pathways, as well as cAMP-response element binding protein (CREB) signaling [68]. At the same time, Que can also alleviate the phototoxicity mediated by photobleaching products and enhance the phagocytosis capacity of RPE cells for photoreceptors’ outer segments. In oxidative stress, lipid peroxidation produces harmful metabolites that accelerate the death of RPE cells, such as malondialdehyde and HNE. Malondialdehyde induces RPE cell junction disruption and autophagy dysfunction [121]. HNE reacts with many cellular proteins, leading to several cytopathological effects, such as the inhibition of nucleic acid and protein synthesis, decreased enzyme activity, cell cycle arrest, and apoptosis [122]. One study showed that Que can protect ARPE-19 cells from the toxicity of HNE and dampen the inflammatory response in stressed cell cultures [123]. Encouragingly, Que-3-O-arabinofuranoside exerts a preventive effect on blue light-induced oxidative damage in RPE cells [124]. Therefore, it can be concluded that Que has potent efficacy in alleviating oxidative stress in AMD models.

The protective effect of Que on neuronal cells in AMD is also reflected in its ability to alleviate inflammation and prevent the apoptosis of retinal cells and neovascularization. This may reverse the key driving step of photoreceptor damage in AMD patients [142]. Que can down-regulate the expression of Bax, cleaved-caspase3, and cleaved PARP and up-regulate the expression of BCL-2 through inhibiting the PI3K/pAKT pathway, thereby protecting NaIO_3_-induced ARE-19 cell apoptosis [91,143]. Que can significantly reduce the expression of inflammatory and apoptotic biomarkers in RPE cells through the KEAP1/NRF2/ARE pathway [125]. In view of these beneficial biological activities, Que may become a promising therapeutic agent for AMD.

Mounting evidence suggests that the epithelial–mesenchymal transition (EMT) of the RPE significantly contributes to subretinal fibrosis, which is the end stage of AMD. EMT leads to the transformation of RPE cells to mesenchymal cells. This process involves the dissociation of cell–cell adhesions, disruption of cell polarity, and remodeling of the extracellular matrix [129,144]. The movement and contractility of mesenchymal cells allow them to invade and migrate in pathological conditions to facilitate tissue fibrosis [145]. These processes can destroy the highly organized anatomical layers and tightly coordinated cellular interactions in the retina, leading to profound vision loss. Isorhamnetin, a metabolite of Que in the liver, has been shown in recent research to alleviate dry AMD-like pathological changes in C57BL/6 mice retina and, additionally, inhibit the migration of Ox-LDL-treated ARPE-19 cells through inhibiting the NRF2-dependent AKT/GSK-3β pathway [129]. These findings suggest its potential to repress the EMT processes both in vivo and in vitro, opening new possibilities for the development of novel treatment directions for AMD. Another study shows that the occurrence of dry AMD may be attributed to lysosomal clearance defects in the RPE, which arise from the diminish of bA3/A1-crystallin [146]. This protein localizes to the lysosomal lumen and plays a crucial role in recruiting the mTOR kinase complex 1 signaling platform on the lysosomal surface [147,148]. Consequently, this leads to impaired lysosomal clearance functions, including phagocytosis and autophagy. In response, RPE cells attempt to mitigate the resulting stress through reversible Type 2 EMT, a process marked by the loss of key epithelial functions while gaining resistance to cell death. It is widely known that mTORC1 serves as a pivotal cellular kinase that integrates major signaling pathways, thereby regulating anabolic and catabolic processes such as autophagy and lysosomal biogenesis [149]. In this process, the activation of TFEB plays a crucial role in mediating the enhancement of autophagic and lysosomal clearance capacity [150]. However, active mTORC1 phosphorylates TFEB, leading to its inactivation and the inhibition of nuclear translocation [151]. As previously discussed, Que can enhance autophagy via multiple pathways, including acting as a direct inhibitor of mTOR and promoting TFEB translocation. Therefore, Que may reverse EMT by modulating lysosomal function to enhance autophagy.

An increasing amount of studies have demonstrated the subtlety of combining polyunsaturated fatty acids (PUFAs) with Que. Conjugates of Que with linoleic or α-linolenic acid, formed using classical chemical techniques or enzymatic reactions, are potent lipophilic antioxidants (Que-3-LA and Que-7-ALA) [131]. They induce a stronger anti-apoptosis effect on ARPE-19 cells compared to parent polyphenol Que [131]. Que-3-LA and Que-7-ALA display substantial protection against A2E toxicity in ARPE-19 cells via anti-carbonyl stress activity. However, the position of PUFAs on the Que core, rather than the type of polyphenol, seems to be more determinative of the antioxidant effect.

Senescent changes in retinal tissue, contemporaneous with immune aging in both innate and adaptive cells, have emerged as pathological contributors to AMD [152]. Senescent RPE cells can accelerate pathological neovascularization. Researchers propose that clearing these aging cells and matrix may serve as a novel therapeutic strategy for AMD. Dasatinib plus Que can specifically kill senescent cells without destroying young cells. In previous studies, the combination of dasatinib plus Que has been used to treat age-dependent hepatic steatosis, fibrotic pulmonary disease, and neural disorders [153,154,155]. Remarkable therapeutic effects have been achieved in these experiments related to cellular senescence. In particular, this compound preparation also has therapeutic potential in age-related retinopathy. The number of senescent cells was increased in a laser-induced CNV rat model. Intravitreal injections of dasatinib combined with Que reduce the expression of p16, a senescence marker in ARPE-19 cells, and alleviate neovascularization [128]. This makes us look forward to the emergence of more effective drug combinations containing Que.

### 4.2. Retinitis Pigmentosa

**Figure 3:**
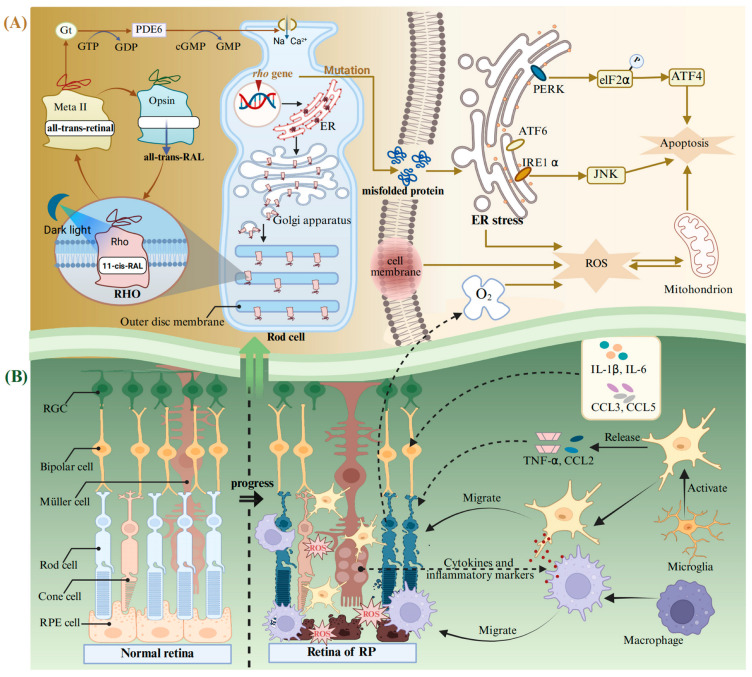
The molecular mechanism of pathological changes in RP. (**A**) Production and circulation of RHO in a normal rod cell, as well as abnormal responses triggered by mutations in the Rho gene during RP. (**B**) The normal retina and its changes during RP. Abbreviations: Gt, G protein transducing; PDE6, a cyclic GMP phosphodiesterase; Meta II, metarhodopsin II; RHO, rhodopsin; ER, endoplasmic reticulum; eIF2α, eIF2kinase; ATF, activating transcription factor; IRE1α, inositol-requiring enzyme-1 alpha; PERK, protein kinase R-like ER kinase; RGC, retinal ganglion cell; RPE cell, retinal pigment epithelial cell; RP, retinitis pigmentosa; CCL, the C-C motif chemokine ligand. Created with BioRender.com.

RP is a heterogeneous group of degenerative retinopathies caused by genetic mutations. The main feature of RP is the loss of the visual field and scotopic vision, accompanied by an intraretinal pigmentation called bone spicules, and, finally, complete blindness [156]. It is reported that the onset ratio of RP is relatively low at young ages and is linearly increased with age until 65 years [157]. RP may present as an isolated condition, referred to as non-syndromic RP, without any additional clinical manifestations, or it may occur as part of syndromic or systemic conditions associated with other neurosensory disorders, developmental abnormalities, or complex clinical phenotypes [156,158]. RP exhibits a high degree of genetic heterogeneity, with mutations in over 100 genes identified as causative factors. To date, approximately 3100 mutations have been documented in these pathogenic genes [158], wherein the mutations in the Rhodopsin (*Rho*) gene are the main cause of autosomal dominant RP blindness [132]. RHO belongs to the G protein coupled receptor superfamily and is the main protein in the outer disc membrane of rod photoreceptors. It can convert dim light signals into a neural impulse, initiating the perceiving biological processes stimulated by dark light in low-intensity light detection or night vision [133,159]. RHO is composed of the ligand-free opsin and 11-cis-retinal bound to its the retinal-binding pocket (Figure 3A). The incident light photon induces the isomerization of 11-cis-retinal to all-trans-retinal, promoting the transformation of RHO conformation into a receptor-activated state called the metarhodopsin II (Meta II) [160,161,162]. Meta II activates the G protein transducin (Gt) and a cyclic GMP phosphodiesterase (PDE6) to catalyze GMP hydrolysis and the exchange between GDP and GTP, resulting in changes in the flux of Na^+^ and Ca^2+^, as well as transmission of chemical signals in rod-shaped cells [163]. After that, the all-trans-retinal is released from the retinal-binding pocket and subsequently converted back to 11-cis-retinal within the visual cycle. The regenerated 11-cis-retinal combines with the ligand-free opsin again and undergoes the above process in a cyclic manner [164].

*Rho* is highly prone to spontaneous mutations, causing misfolding and retention in the ER (Figure 3A) [165]. This leads to an uncontrolled activation of ER stress, which is mediated by the inositol-requiring enzyme-1 alpha (IRE1α), the eIF2 kinase (elF2α), and the activating transcription factor (ATF) 6 [166]. The ER stress pathway ultimately results in apoptosis of photoreceptor cells through PERK/elF2α/ATF4 and IRE1α/ JNK [167]. It can trigger an inflammatory response and activate neuralgia in the retina. Sustained inflammatory response will exacerbate oxidative stress and induce photoreceptor apoptosis [142]. Rods are the main consumers of O_2_ in the retina [168]. The death of rods will lead to the accumulation of large quantities of O_2_ in the retina, exacerbating the process of RP [168]. This will lead to the occurrence and development of the three phases of RP (Figure 3B): (i) Photoreceptors suffer stress and the rods shorten. (ii) Deaths of photoreceptors and other retinal neurons take place, and Müller cells fill these vacancies and form glial fibrotic walls throughout the retina. (iii) Remodeling of the neuronal, glial, and vascular occurs [169,170]. Microglia can be activated after the oxidative damage to macromolecules (lipids and DNA) in the retina [156]. In addition, the retinal Müller glial cells guide the microglia and macrophages to migrate to the outer retina, facilitating the clearance of dying photoreceptors through cytokine and inflammatory mediator secretion (Figure 3B) [171,172]. Simultaneously, the activated microglia exhibit uncontrolled secretion of pro-inflammatory chemokines, including the C-C motif chemokine ligand (CCL) 2 and TNF-α [173]. At the same time, other inflammatory mediators also increase, including cytokines IL-1β and IL-6 and chemokines CCL3 and CCL4 [174,175].

In this context, many drugs have been developed to target *Rho* mutated genes and RHO misfolding proteins. Several types of compounds have been used to counteract *Rho* mutation defects, like 11-cis-retinal, valproic acid, and some antioxidants [81]. As demonstrated previously, flavonoids can interact with the opsin apoprotein [133]. Que could bind to the ligand-free opsin accommodated within the retinal-binding pocket and act as a structural stabilizer [164]. One study shows that treating the *G90v* mutant opsin with Que can increase its chemical stability and chromophore regeneration rate [81]. Simultaneously, Que can slow down the Meta II decay process of *G90v* mutant opsin and induce a clear change in the kinetics mode of the G-protein transducin activation [81]. Moreover, Que and Que-3-rhamnoside significantly enhance the stability of opsin, presumably by imparting structural rigidity and facilitating receptor self-association within biological membranes [133]. Another study shows that Que could interact within the orthosteric binding pocket of *P23h* RHO, thereby shifting the conformation of its N-terminal loop to a state more closely resembling the wild type [132]. These biological activities can inhibit the signaling of the unfolded protein response and the expression of oxidative stress-related markers. On the other hand, neuroinflammation is a critical hallmark of RP pathology. Exposure to strong white light caused photoreceptor degeneration in Tvrm4 mice heterozygous for RP with the *I307n* dominant mutation of RHO [82]. Then, treatment with Que could maintain the retinal function and alleviate photoreceptor cell death by inhibiting the neuroinflammation [82]. Collectively, these findings suggest that Que may be developed into a novel therapeutic remedy for RP.

### 4.3. Glaucoma

**Figure 4:**
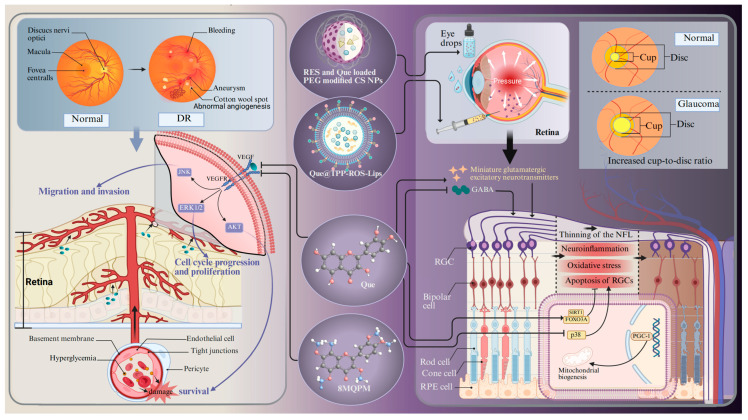
The molecular mechanism of Que in the treatment of DR and glaucoma. Abbreviations: DR, diabetic retinopathy; RES- and Que-loaded PEG-modified CS NPs, Que loaded in chitosan nanoparticles modified by natural antioxidant resveratrol; QUE@TPP-ROS-Lips, encapsulated Que in ROS-responsive mitochondria-targeted liposomes; 8MQPM, a permethylated form of Que; NFL, nerve fibers layer; FOXO3A, forkhead box O3a; PGC-1, peroxisome proliferator-activated receptor γ coactivator-1; GABA, γ-aminobutyric acid. Created with BioRender.com.

Glaucoma is a chronically progressive disease that leads to the thinning of the retinal nerve fiber layer, RGC loss, and an increased cup-to-disc ratio [83]. It is one of the predominant diseases causing irreversible blindness. More than 60 million people worldwide are suffering from glaucoma [176]. Aging is one of the most serious risk factors for glaucoma, with a high prevalence in individuals aged 60 years and older [177]. The potential mechanisms contributing to cellular senescence in glaucoma encompass oxidative stress, mitochondrial dysfunction, DNA damage, epigenetic modifications, and defective autophagy. These characteristics interact and create a stable network that sustains the cell senescent state. Aging and stress appear to induce the accumulation of senescent trabecular meshwork cells, RGCs, and vascular endothelial cells, which are believed to play a role in the development of glaucoma-related pathologies [177]. Free radicals are involved in aging and are generated in the retina as a result of ischemia, leading to oxidation of the surrounding neurons and subsequently inducing glaucoma-related cell death via apoptosis [177]. It is difficult for doctors to make an early diagnosis because glaucoma is insidious and does not show any obvious symptoms until a relatively late stage [178]. Glaucoma is usually treated with daily eye drops to lower the intraocular pressure, but the therapeutic results are barely satisfactory [179]. The precorneal contact time and ocular bioavailability of eye drops are contingent upon the barrier function of the ocular surface, the blinking process, adhesion of drops, and tear production [180]. Moreover, these eye drops often induce significant side effects, such as adverse dermatological effects in the ocular and periocular areas (e.g., contact dermatitis and mucous membrane pemphigoid) [181,182]. In addition, benzalkonium chloride, the preservative included in hypotensive eye drops, can cause conjunctival metaplasia, corneal damage, and disruption of the tear film [183]. Therefore, it is imperative to search for alternative neuroprotective drugs and drug delivery systems. An in vivo experiment shows that Que can improve the survival and function of RGCs in a chronic glaucoma rat model (Figure 4) [134]. The underlying mechanism should be ascribed to the increased transportation efficiency of the inhibitory neurotransmitter γ-aminobutyric acid to RGCs. Moreover, Que treatment inhibits the release of glutamatergic excitatory neurotransmitters and dampens the excitability of RGCs [134]. Another in vitro experiment shows that Que alleviates hypoxia-induced RGC injury through improving mitochondrial function [93]. Apart from the neuroprotective effects, experimental evidence also shows that Que can also increase the expression of genes related to mitochondrial biogenesis (e.g., peroxisome proliferator-activated receptor γ coactivator-1 (PGC-1α) and mitochondrial transcription factor A) in glaucoma cybrids [184]. All of these experiments suggest that Que has tremendous potential for treating glaucoma.

### 4.4. Diabetic Retinopathy

DR is a critical microvascular complication of diabetes mellitus, characterized by RNV, chronic inflammation, disorders of glucolipid metabolism, and immune response as its hallmarks. It acts as the prevailing cause of blindness among the working population in developed countries, and more than 100 million people worldwide are suffering from DR [185]. Diabetic macular oedema is one of the major complications of DR. Macular vascular pathology leading to exudates and edema at the macula is most common in older patients [186]. The diagnosis of DR mainly lies in the detection of fundus microangiopathy on the basis of diabetes mellitus. Based on this, it can be divided into non-proliferative DR and proliferative DR. Non-proliferative DR is characterized by microaneurysms, intraretinal hemorrhage, hard exudate, and cotton wool spots. In contrast, proliferative DR is characterized by fundus and iris neovascularization with vitreous hemorrhage [187]. The neurodegeneration of RGCs and the activation of glial cells may be some of the earliest events in the pathogenesis of DR. These events occur before the onset of vascular pathology and contribute to the occurrence and progression of DR [188]. Thus far, the clinical management of DR still faces tremendous challenges. Despite the fact that laser therapy and vitrectomy surgery drugs have afforded definite benefits for DR patients, they cannot halt disease progression or reverse vision loss [189]. Therefore, a novel medication is needed to alleviate the symptoms of DR.

Given the pivotal role of RNV in the advancement of DR, the primary objective of clinical management and fundamental research in DR is the continuous monitoring of retinal vascular lesions [190]. VEGF is one of the most dominant causative factors in DR pathology, and inhibiting VEGF expression acts as an efficacious strategy to treat DR. One study shows that Que can inhibit VEGF expression through suppressing pAKT and MAPK [93]. In addition, a hypermethylated derivative of Que, 8MQP (Figure 4), is endowed with a strong capacity to inhibit angiogenesis. It can suppress the activation of VEGFR2 downstream signaling pathways (VEGFR2/AKT, VEGFR2/MEK/JNK, VEGFR2/MEK/ERK1/2), thereby mitigating the migration of vascular endothelial cells [79]. In particular, the evidence suggests that Que can inhibit the viability, migration, and tube formation of human retinal microvascular endothelial cells under high glucose stimulation by inhibiting the NLRP3 inflammasome and autophagy signaling pathways, which play an important role in the pathogenesis of DR [190]. Surprisingly, in vivo experiments demonstrate that Que markedly increases the thickness of the retinal cell layer and the number of RGCs by inhibiting the HMGB1/TLR4/NF-κB/NLRP3 inflammasome/IL-1β/IL-18 axis, suppressing VEGF and sICAM-1 secretion, and promoting brain-derived neurotrophic factor secretion through the up-regulation of HO-1 [140]. Another in vivo experiment demonstrates that Que relieved neuroinflammation and photoreceptor apoptosis in a streptozotocin-induced DR rat model [57,137]. The anti-inflammatory effect mediated by Que is also verified by behavioral assessment and antioxidant assays in a zebrafish DR model [138]. Although studies have implicated that DR is caused by chronic hyperglycemia, the exact pathogenesis remains unknown. A multitude of studies have demonstrated that the gut microbiota is involved in retinal neurodegeneration and retinal inflammatory processes in DR through affecting the metabolism of glucose, antioxidant enzymes, lipids, and entero–insulin secretion [190]. However, Que can reverse these changes by inhibiting intestinal dysbiosis in a model of hyperglycemic rats induced by streptozotocin injection and high-fat diet intervention [141].

## 5. New Technology and Methods for Drug Delivery

In pharmacological investigations, Que can be administered orally or in the form of peritoneal or intravenous injections. Although Que can pass through the blood–retinal barrier after oral or systemic administration due to its liposolubility, the investigation on the pharmacokinetics of Que in humans suggests very poor oral bio-availability after a single oral dose (~2%) [25]. The estimated absorption rate of Que glucoside, the naturally occurring form of Que, ranges from 3% to 17% in healthy individuals receiving a 100 mg oral dose [25]. Que exhibits limited oral bioavailability, as only 20% of the ingested dose is distributed to the eye, owing to its hydrophobic characteristics [11,12]. Particularly, Que metabolites appear in plasma after 30 min of ingestion, but a significant amount is excreted over a 24-h period. These findings indicate the low absorption, substantial first-pass metabolism, extensive metabolism, rapid elimination, and a short half-life of Que. Accordingly, Que has low absorption, substantial first-pass metabolism, extensive metabolism, and rapid elimination. In the eye tissue, Que can also be delivered through topical drops or intravitreal injections to increase its concentration. Nevertheless, the restricted solubility of Que, in conjunction with its insufficient retention and targeting capabilities, markedly impedes its efficacy in the treatment of eye fundus diseases [191,192]. Additionally, frequent intravitreal injections carry the risks of eye damage and potential infections. In particular, there are also enzymes in the eyes that can react with Que. In vitro experiments show that Que can enter the lens from surrounding artificial aqueous humor. In the lens, enzymes can metabolize Que to 3′-O-methyl Que [193]. In this context, pharmacokinetic factors determine the required high doses and rates needed for drug action. Differences in administration methods and derivatives types may exert impacts on the amount, efficiency, and chemical form of Que that ultimately reaches the retina tissue. Consequently, more mechanism research is necessary to characterize the exact metabolism and bioavailability of Que in retinal tissue.

**Figure 5:**
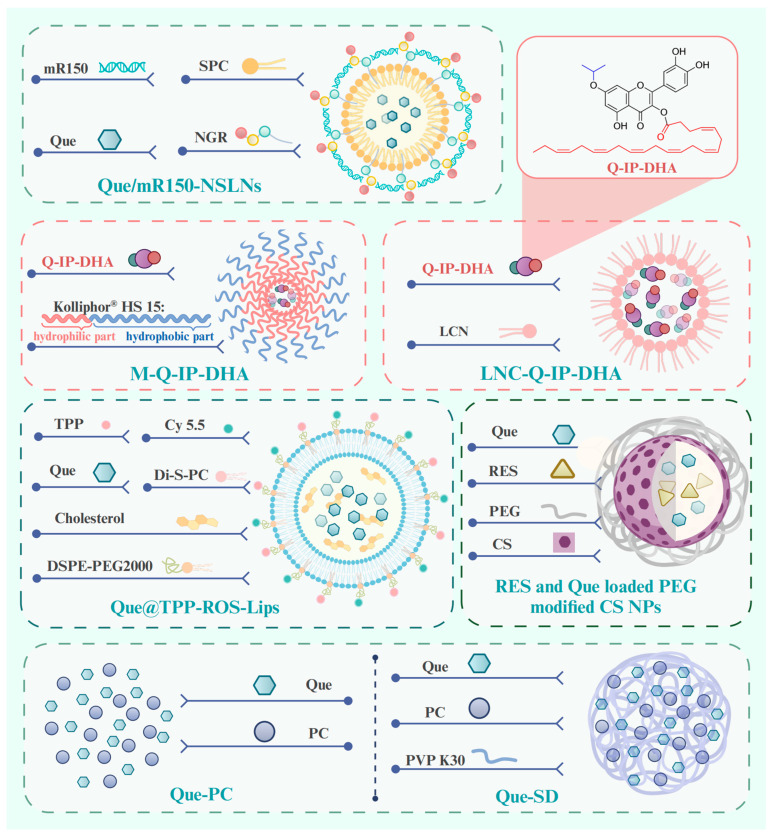
Structure of pre-clinical drug delivery methods for Que in the treatment of age-related retinopathy. Abbreviations: mR150, microRNA-150; SPC, Soy phosphatidylholine; NGR, asparagine–glycine–arginine; Que/mR150-NSLNs, asparagine–glycine–arginine peptide-modified solid lipid nanoparticles for co-delivery of microRNA-150 and Que; Q-IP-DHA, 3-O-docosahexaenoicacid-7-O-isopropyl-quercetin; M-Q-IP-DHA, micelles-Q-IP-DHA; LNC-Q-IP-DHA, lipid nanocapsules-Q-IP-DHA; TPP, triphenylphosphonium; Di-S-PC, a liposome formulation composed of thioether phosphatidylcholines; Que@TPP-ROS-Lips, Que in ROS-responsive mitochondria-targeted liposomes; RES, resveratrol; CS: chitosan; PEG, polyethylene glycol; NPs, nanoparticles; Que-PC, phospholipid complex of Que; Que-SD, solid dispersion of Que-PC. Created with BioRender.com.

To circumvent the limited bioavailability of Que, researchers have investigated new technologies and methods for the drug delivery of Que in degenerative retinopathies. We comprehensively reviewed new technologies and methods for ocular posterior drug delivery based on nanostructures, including lipid nanoparticles (NPs), chitosan (CS), solid dispersion (SD), liposomes and micelles, and hydrogels (Figure 5) [194]. We describe their structure and mechanism of action with higher drug encapsulation efficiency (DEE) and drug loading efficiency, prolonged retention, controlled drug release, and therapeutic effects, showing promise for the clinical application of Que in age-related retinopathy.

### 5.1. Lipid-Based Delivery Systems

Lipid-based delivery systems are the most common and well-studied nanodelivery systems as nanostructure vehicles of Que in ocular posterior drug delivery, including lipid NPs, liposomes, and nanoemulsions. They have garnered extensive attention due to their low toxicity and good biocompatibility and biodegradability, as well as their capability to transport both hydrophobic and hydrophilic compounds [127,195].

#### 5.1.1. Lipid Nanoparticle Delivery Systems

Lipid NPs are spherical structures composed of a solid lipid core matrix or a mixture of solid and liquid lipids which are stabilized by surfactants and can be divided into solid lipid NPs (SLNs) and nanostructured lipid carriers (NLCs) [196]. SLNs are crafted from lipids that exhibit solidity at either body or room temperature [196]. Li et al. prepared SLNs co-loaded with Que and microRNA-150 by the film–ultrasonic method and modified them with an asparagine–glycine–arginine (NGR) peptide (Que/mR150-NSLNs) (Figure 5). The Que/mR150-NSLNs had a particle size of 209 nm and a DEE of 85.25 ± 1.29%. It is noteworthy that the SLN carriers evaluated in this study included cationic lipid (dioctadecyl dimethyl ammonium bromide), which facilitates the encapsulation of negatively charged nucleic acid drugs into SLNs through its cationic charge [52,127]. After intravitreal injection, the NGR can specifically target CD13 receptors, which are overexpressed on endothelial cells during abnormal angiogenesis [130]. Subsequently, microRNA-150 can suppress choroidal angiogenesis through inhibiting the expression of C-X-C motif chemokine receptor 4 at the post-transcriptional level. The Que/mR150-NSLNs exhibited strong bioavailability to endothelial cells and inhibited CNV in a laser photocoagulation-induced CNV mouse model for up to two weeks without causing obvious toxic effects. Moreover, they significantly enhanced the uptake of microRNA-150 compared with free microRNA-150 or NPs without the peptide. A significant benefit of SLNs is their markedly restricted drug mobility, which prolongs the release of the drug, which is a crucial prerequisite in facilitating the escape of the drug from the vitreous humor and its migration to the retina. However, its most major disadvantage is the low DEE.

NLCs are derived from SLNs and are composed of a mixture of solid and liquid lipids. In addition to exhibiting the beneficial characteristics of SLNs, they also address the significant drawback of SLNs, namely the low DEE [197]. The resulting matrix of liquid and solid lipids exhibits significant imperfections in the crystal lattice, allowing for enough space to accommodate drug molecules, thereby enhancing the drug loading capacity and providing greater flexibility for modulating drug release [198,199]. Yibin Yu et al. [200] prepared a NLC loaded with Que (QN-NLC) using melt–emulsification combined with the ultra-sonication technique. It was characterized by narrow size distribution and high DEE of 97.14% with a mean particle size of 75.54 nm.

#### 5.1.2. Liposomes Delivery Systems

Liposomes are self-closed, phospholipid spherical vesicles constructed from one or more concentric lipid bilayers (phospholipids and cholesterol) which enclose an internal aqueous phase [196,201]. As a well-established and extensively utilized nanoplatform, liposomes possess several recognized advantages, such as inherent biocompatibility, biodegradation, nontoxicity, the flexibility to conjugate various ligands, and high DEE of hydrophobic drugs [196,202,203]. For these reasons, liposomes are capable of prolonging the duration of drug treatment effects as well as maintaining drug levels within the posterior segment of the eye [204]. In a recent study, researchers encapsulate the hydrophobic compound Que in ROS-responsive mitochondria-targeted liposomes (Que@TPP-ROS-Lips, DEE of 90.2% ± 4.67%) (Figure 1) to achieve controlled and specific drug release [135]. Di-S-PC, a liposome formulation composed of thioether phosphatidylcholines in Que@TPP-ROS-Lips, exhibits stimuli-responsive properties and favorable biocompatibility. It is employed as a drug carrier specifically targeting oxidative stress environments. Simultaneously, triphenylphosphonium (TPP) is incorporated into the liposomes to target mitochondria, a critical source of ROS in glaucoma, while DSPE-PEG2000 is added to increase the liposome stability. The cholesterol contributes to stabilizing fluid bilayers, thereby reducing the leakage of contents from liposomes [205]. In vitro experiments have shown that the ability of Que@TPP-ROS-Lips to escape from lysosomes after being internalized by R28 cells is an important ability for drug subcellular localization. In vivo studies further reveal that, following intravitreal injection, Que@TPP-ROS-Lips primarily localize in the ganglion cell layer and remain in the retina with a constant particle size for at least 2 weeks. Concurrently, Que@TPP-ROS-Lips exert potent anti-neuroinflammatory and anti-apoptotic effects on both R28 retinal cells (retinal precursor cells) and a rat model of retinal ischemia–reperfusion [135]. The deep-rooted mechanism by which it exerts this effect is through the regulation of the SIRT1/FOXO3A and p38 MAPK signaling pathways. It is necessary to test it in future studies in chronic models of glaucoma, DR, and AMD.

#### 5.1.3. Nanoemulsion Delivery Systems

Nanoemulsions are dispersed oil droplets, which are divided into water-in-oil (W/O) and oil-in-water (O/W) droplets. The droplets are stabilized by an amphiphilic surfactant [206]. The oil nanodroplets can serve as effective reservoirs for solubilizing a range of lipophilic ingredients, thereby protecting them from degradation caused by external factors, such as oxidation, pH, and hydrolysis [206]. Due to their high drug loading capacity, physical stability, and ease of production and sterilization, as well as compatibility with living tissues, O/W nanoemulsion systems have been extensively pursued for hydrophobic pharmaceutical compounds [207,208]. Specially, a O/W nanoemulsion system loaded with Que exhibited a remarkable drug entrapment efficiency of 100% and good stability against phase separation and storage at 4 °C for 3 months [209]. Furthermore, the low viscoelastic properties of O/W nanoemulsions facilitate their straightforward injection via minute needles [206]. Consequently, they may be suitably employed for the targeted delivery of hydrophilic drugs through intravitreal injection [210]. Given that oils have a propensity to solubilize hydrophobic drugs, the solubility of Que in the oil component is significant. Another study assessed the effectiveness of Que delivered via a novel ocular self-microemulsifying drug delivery system (SMEDDS). By utilizing oils and surfactants (propylene glycol and Tween 80), the SMEDDS formed microemulsions that enhanced solubilization, absorption, and dissolution in the gastrointestinal tract, thereby improving stabilizing labile molecules and oral bioavailability [211]. The findings indicate that the SMEDDS achieved 99.85% entrapment efficiency, 12.37 nm droplet size, and 89.55% in-vitro drug release in pH 7.4 phosphate buffer [212]. SMEDDSs have been studied for various eye diseases, including dry eye syndrome, glaucoma, uveitis, and AMD [212]. It seems clear that a specific study of SMEDDSs and nanoemulsions loaded with Que formulations should be performed in in vivo and animal experiments.

### 5.2. Hydrogel-Based Delivery Systems

Hydrogels are 3D polymeric networks with high water content (>90%) that can be easily customized and formulated into particles with various sizes and shapes [213]. Their rheological properties can be well characterized via the oscillation mode on a rheometer (e.g., stress, strain, temperature, etc.), which facilitates the development of novel drug delivery platforms [214,215]. Hydrogels are considered ideal drug carriers for regulated and sustained release at sites of interest, as well as for the assessment of treatment efficacy. Previously, the utilization of thermoresponsive in situ gels as ophthalmic drug delivery systems has been reported. They are a solution at the temperatures outside the eye and become a gel when exposed to the eye’s temperature. This characteristic enables Que to be loaded onto a hydrogel in a liquid state, which can be readily administered and solidified upon application [216]. Purnama et al. developed formulations of thermoresponsive Que nanoemulgels (T-QNE-Gs) that addressed the low viscosity and spreadability of nanoemulsions [210]. These were prepared by incorporating gels consisting of Pluronic F127 to Pluronic F68 at an optimum ratio (2:1), with a fixed concentration of hydroxypropyl methylcellulose, into a Que nanoemulsion concentrate. T-QNE-Gs can flow properly and be injected into the vitreous humor easily at room temperature (27 ± 1 °C). At the temperature of the posterior eye segment (35 ± 1 °C), the solution becomes gel-like and maintains its state in the simulated vitreous humor for a longer time (20 h) than a Que nanoemulsion (5 min) [210]. In a separate study, a thermosensitive gel loaded with Que NPS and epigallocatechin gallate was formulated with the objective of increasing the antioxidant and intracellular ROS inhibition effects in HCE cells [217]. Que was loaded into Poly Lactic-co-Glycolic Acid NPs using a solvent displacement method. The hyaluronic acid adsorbed on the surface of Que-loaded Poly Lactic-co-Glycolic Acid NPs is a glycosaminoglycan possessing a negative charge and high viscosity. It serves as a stabilizer and a mucoadhesive polymer that can facilitate the adhesion of NPs to the ocular surface. In another study, QN-NLC-Gel was created by the addition of QN-NLC to an F127/CMCS hydrogel using the swelling method, thereby enhancing its retention capability on the ocular surface [200]. The F127/CMCS hydrogel was both temperature-responsive and pH-responsive, having a high swelling ratio under 35 °C and pH 7.4 and a stronger prolonged release characteristic (80.52% after 72 h) compared to a Que solution (98.38% after 12 h).

### 5.3. Chitosan-Based Delivery Systems

Topical ophthalmic formulations are generally administered more than once a day at relatively high drug concentrations to maintain the necessary therapeutic levels of a drug at a target site [218]. However, mucoadhesive polymeric drug delivery systems can increase the residence time of eye drops, resulting in a higher drug flux through the absorbing tissues and reducing the administration frequency. Subramanian et al. [136] developed a mucoadhesive polymeric drug delivery system that uses CS-loaded resveratrol (RES) and Que (Figure 5), both of which are natural antioxidants, and then prepares NPs using the ionic gelation method. The CS is non-toxic and biocompatible and exhibits pseudoplastic and viscoelastic qualities in solution, helping to prolong the delivery of the loaded drug [219]. In addition, the incorporation of polyethylene glycol (PEG) in the formulation can improve the biocompatibility and stability. Dropping RES- and Que-loaded PEG-modified CS NPs onto the eye surface of normotensive rabbits can improve corneal permeation and consistently lower the intraocular pressure [136].

### 5.4. Gold-Based Delivery Systems

The utilization of high-density gold or inorganic NPs has the potential to disrupt cell–cell interactions, thus facilitating enhanced drug delivery [220]. An in situ ophthalmic tethered gold yarnball (GY) was synthesized using the seed-growth method within the defects of an AgCl cube, which served the dual purpose of acting as an ocular retention agent and a Que reservoir, thus overcoming the issue of low fundus drug retention [220]. After intravitreal injection in a mouse, the gold nanoparticle-Que complexes (QC@GYs) increased retinal cell leakage and internal limiting membrane permeability into the intraretinal tissue. This observation suggests that the GY has greater Que loading efficiency (≈40%). These favorable drug-carrying results may be attributed to hydrophobic interactions between the Que molecules and the metal surface of the GY, as well as the increased surface area for adhesion. Subsequently, the GY cavities provide a network of interconnected voids or channels within the particle structure which function as drug reservoirs, exhibiting sustained-release properties from the GYs for a minimum of 15 days. This preservation of retinal morphology and function optimizes the therapeutic impact while reducing the necessity for frequent intraocular administration.

### 5.5. Micelle-Based Delivery Systems

The nano-sized micelles are amphiphilic molecules or block copolymers that are capable of self-assembly into organized core–shell structures (comprising a hydrophobic core and a hydrophilic shell) in aqueous media at concentrations exceeding their critical micellar concentrations [221]. The presence of Que loaded in micelles is less recognized by macrophages, which results in an increase in its half-life in the bloodstream [222]. A new innovative lipophenolic molecule, named 3-O-DHA-7-O-isopropyl-quercetin (Q-IP-DHA), has been developed based on an alkyl polyphenol core that is linked to a PUFA part. Q-IP-DHA provides an isopropyl Que moiety that is able to reduce oxidative stress. It is covalently linked to docosahexaenoic acid (DHA), a PUFA proven to enhance the bioavailability of Que by helping its derivatives pass through retinal cell membranes and across the RPE cell barrier [223]. Given that Q-IP-DHA is a poorly water-soluble molecule classified as a BCS class IV drug, researchers added an intravenous formulation with micelles (Kolliphor^®^ HS 15) (M-Q-IP-DHA) (Figure 5) and an oral formulation using lipid nanocapsules (LNC-Q-IP-DHA) (Figure 5) to improve the solubility and bioavailability of Q-IP-DHA [130]. M-Q-IP-DHA (mean size of 16 nm, drug loading of 95%) and LNC-Q-IP-DHA (25 nm, 96%) are allowed for intravenous and oral administration, respectively, in a mice model with light-induced photoreceptors degeneration and BALB/c mice with dry AMD. Kolliphor^®^ HS 15 is a non-ionic, low-toxicity, amphiphilic surfactant composed of 70% of ethoxylated mono- and di-ester components. M-Q-IP-DHA is dilution stable, which restricts the possible re-precipitation rate of Q-IP-DHA in the blood to 4.5% of the administered dose during blood injection. LNC-Q-IP-DHA significantly improves photoreceptor protection compared to Q-IP-DHA in a dose-dependent manner (25, 30, 50, 75, and 100 mg/kg), reflecting its enhancement of oily solubilization and permeability. In this context, the delivery of Q-IP-DHA by micelles and lipid nanocapsules may provide an efficient strategy for managing age-related retinopathy.

### 5.6. Solid Dispersion-Based Delivery Systems

Enhancement of the lipophilicity of Que by modifying its structure is a useful method to improve its absorption and facilitate its formulation for in vivo administration [126]. For example, in another study, Que was combined with the active ingredient phospholipid complex (PC) extracted from Chinese herbal medicine to form Que-PC (Figure 5) [126]. Que-PC has a three-fold increase in water solubility and a 734-fold increase in lipid solubility. Que-PC can produce stronger pharmacological effects, owing to the longer absorption and action time compared to Que. However, although the formation of PC significantly improves the solubility of Que, the hydrophobicity of phospholipid complexes leads to a poor dispersion rate and limited bioavailability of Que. Researchers then combined SD, specifically, polyvinylpyrrolidone, with Que-PC to form Que-SD (Figure 5). Solid dispersion techniques can be employed to disperse Que-PC in an inert carrier excipient in the solid form, thus addressing the problems related to solubility and permeability. This technique allows for the complete removal of drug crystallinity and molecular dispersion of a poorly soluble drug in a hydrophilic polymeric carrier [224]. Que-SD has improved physicochemical and pharmacokinetic properties in comparison to Que and Que-PC and exhibits more potent protective effects on retinal oxidative injury in vivo [119].

## 6. Conclusions and Future Perspectives

Chronological aging represents the single greatest risk factor for human disease [225]. During this process, the retina is at an increased risk of promoting various age-related retinopathies [59,226]. By regulating oxidative stress and related cellular responses, which are regarded as critical processes involved in the degenerative processes and metabolic disorders of retinal systems, Que has been validated to be effective in treating a variety of age-related retinopathies. The versatile characteristics of Que make it a beacon of hope in the quest for improved disease management and therapy for age-related retinopathy. However, the application of Que therapy is confronted with various limitations. The low water-solubility and absorption rate, poor bioavailability, rapid clearance, metabolism, and enzymatic degradation largely prevent the clinical usage of Que for age-related retinopathy. With the development of pharmacological science, novel biomaterials, drug formulations, and delivery techniques are needed to resolve these problems. Researchers are endeavoring to create efficient approaches to improve the bioavailability of Que, including structural modifications, the production of pro-drugs, and formulation into NPs. However, due to the modification of its chemical structure, other chemical characteristics of Que are often altered to meet a specific requirement. Developing a unique structural change that can be used in all circumstances is what we hope for. It is noteworthy that more attempts are needed to evaluate the efficiency and toxicity of new delivery systems via large-scale clinical trials. Although Que does not induce serious toxic effects on the retina, some experimental data have reported side effects. As a potential inhibitor of GSH reductase, Que can affect the level of GSH in retinal tissue. Therefore, systematic pharmaceutical toxicology studies of both acute and long-term toxicities are necessary to verify the safety and facilitate the application of Que in ophthalmological practice.

## Figures and Tables

**Table 1:** The therapeutic experiment of Que and its derivatives on age-related retinopathy.

Disease	Drug	Experimental Animal and Cell/Dosage/Mode	Disease Model	Signal Pathways or Molecules/Gene of Action	Curative Effect	Refs.
AMD	Que	ARPE-19 cells (20 µM; 1 h)	IL-1β-induced inflammatory response	MAPK, NF-κB	Ameliorating the inflammatory response.	[67]
	Que	661W (5, 50 µM; 1 h), mice (30 mg/kg/day; 4 days; IP)	Llipopolysaccharide-treated retina inflammation model	ERK/STAT3	Suppresses retinal inflammation and promotes the switch from the M1 to the M2 microglial phenotype.	[70]
	Que	Mice (100 mg/kg; 1 week IP)	NaIO_3_-induced retinal damage model	NRF/PGC-1α/SIRT1	Regulating mitochondrial reactive oxygen species homeostasis.	[91]
	Que	ARPE-19 cells (1.25, 2.5, 5 µM; 1.5 h)	NaIO_3_-induced AMD model	PI3K/AKT, ROS	Inhibiting NaIO_3_-mediated apoptosis.	[91]
	Que-SD	Mice (50, 100, 200 mg/kg; 3 months; IG)	*Nrf2* wild type and *Nrf2* knock-out mice model of dry AMD	NRF2, HO-1, NQO1, GCL	Reducing RPE sediments and Bruch’s membrane thickness, protecting retina from oxidative injury.	[119]
	Que-3-O-arabinofuranoside	ARPE-19 cells (30 µM; 72 h)	Blue light-induced RPE cell death	Cleaved caspase3, Bax, Bcl-2	Inhibiting blue light-induced photooxidation and the intracellular accumulation of A2E.	[124]
	Que	ARPE-19 cells (0.5 µM; 24 h)	Cigarette smoke extract-induced AMD model	KEAP1/NRF2/ARE	Inhibiting inflammation and apoptosis.	[125]
	Que-PC	ARPE-19 cells (200, 400 µM; 6 h)	H_2_O_2_-induced oxidative injury model	NRF2, HO-1, NQO1, GCL	Activating antioxidant defense and preventing ARPE-19 cells from apoptosis.	[126]
	Que/mR150-NSLNs	HUVECs, mice (2 µL, 0.6 µg Que; 2 weeks; IVI)	Laser photocoagulation-induced CNV model	The *Cxcr4* gene, hypoxia inducible factor-1α, and VEGF	Showing strong ability to target the fundus and inhibit CNV.	[127]
	D+Que	Rats (5 µL, 10 ng/µL dasatinib + 50 ng/µL Que; IVI), Mice (3.5 mg/kg dasatinib + 35 mg/kg Que; IP or OG)	Laser-induced CNV rats model, laser-induced CNV mice model	Bcl-2, Bax	Accelerating apoptosis in senescent ARPE-19 cells, alleviating the progression of laser-induced CNV.	[128]
	Isorhamnetin	Mice (10, 20 mg/kg/day; 3 months; OA)	LCZ-induced EMT	NRF2, AKT/GSK-3β	Alleviating dry AMD-like pathological changes and repressing the EMT processes.	[129]
	M-Q-IP-DHA,	Mice (30 mg/kg; 5 days; IV)	Acute light stress-induced photoreceptor degeneration	None	Stabilizing the dilution of Q-IP-DHA, limiting its re-precipitation in the bloodstream to 4.5% of the administered dose.	[130]
	LNC-Q-IP-DHA	Mice (100 mg/kg; 5 days; OA)	Acute light stress-induced photoreceptor degeneration	None	Protecting photoreceptors from light-induced photoreceptors damages efficiently both to oily solubilization and to permeability enhancement.	[130]
	Que-7-ALA, Que-3-LA	ARPE-19 cells (10, 20, 40, 60, 80 µM; 1 h)	H_2_O_2_-induced ROS production	None	Protecting ARPE-19 cells from A2E-induced cell death, possessing antioxidant properties.	[131]
RP	Que	COS-1 cells (1 µM; 48 h)	Plasmids encoding *G90v* mutant transfects COS-1 cells	Metarhodopsin II, Extracellular loop 2	Improving the folding and structural stability of the *G90v* RP mutant.	[81]
	Que	Tvrm4 mice (100 mg/kg/day; 35 days; OA)	*I307n* RHO dominant mutation expresses in Tvrm4 mice	GSH-px, Lactoperoxidase, Myeloperoxidase, NADPH oxidases	Recovering of retinal neurons, slowing retinal degeneration, and inhibiting oxidative stress and apoptosis in retinal tissue.	[82]
	Que	NIH-3T3 cells (10 µM; 4 h), mice (3 injections total at 20 mg/kg; every other day; IP)	*P23h* RHO mutant expresses in NIH-3T3 cells, RHO *P23h*/+ mice, RHO *P23h*/P23 h mice	N-terminal loop	Increasing the conformational stability of *P23h* RHO, improving eye morphology, increasing levels of visual receptors, and recovering visual function.	[132]
	Que, Que-3-rhamnoside	NIH-3T3 cells (1, 10, 100, 250, 500 µM; 1 h)	*P23h* RHO mutant expresses in NIH-3T3 cells	None	Increasing stability of *P23h* RHO folding and membrane targeting, increasing regeneration of the visual pigment, and restoring opsin normal cellular trafficking.	[133]
Glaucoma	Que	Rats (2 µL/week, 10 µM; 4 weeks; IVI)	Paramagnetic polystyrene microbead-induced chronic ocular hypertension in rats	Bcl-2, caspase3	Protection of RGCs by improving mitochondrial function and preventing mitochondria-mediated apoptosis.	[93]
	Que	Rats (2 µL/week, 10 µM; 4 weeks; IVI)	Superior scleral vein electrocoagulation-induced chronic glaucoma	None	Reducing excitotoxicity and glutamatergic excitatory neurotransmission in RGCs, increasing gamma-aminobutyric acid inhibitory neurotransmission.	[134]
	Que@TPP-ROS-Lips	Adherent retinal precursor cell line derived from rat retina (20 µM, 80 µM; 24 h), Rats (4 µL, 20 µM; 1 week, 2 weeks; IVI)	Oxygen–glucose deprivation-induced retinal ischemia–reperfusion. Injection of sterile saline-induced retinal ischemia–reperfusion.	SIRT1/FOXO3A, p38 MAPK, GSH	Attenuating the accumulation of ROS and the reduction in mitochondrial membrane potential caused by RIR injury. Reducing retinal neuroinflammation, oxidative stress, and apoptosis.	[135]
	RES- and Que-loaded PEG-modified CS NPs	Rabbits (50 µL; 8 h; eye drop)	Normotensive rabbit	None	Reducing intraocular pressure in normotensive rabbits effectively and durably.	[136]
DR	Que	Rats (20 mg/kg, 50 mg/kg; 24 weeks; OG)	STZ-induced DR rat model	TNF-α, IL-1β, NF-κB, caspase3, GSH, super oxide dismutase, and catalase	Treatment of DR by alleviating retinal neurodegeneration and oxidative stress.	[57]
	Que	661W cells (0–0.5 h)	VEGF-induced angiogenic signaling pathway in photoreceptors	VEGF, intracellular adhesion molecule 1, cascular adhesion molecule 1, zona occludins, AKT, ERK, and p38	Inhibiting the production of inflammatory proteins, attenuating inflammatory response.	[78]
	8MQPM	HRMECs (25 µL, 4 h)	VEGF stimulates HRMEC to imitate angiogenesis	VEGF receptor 2, AKT, ERK, and the c-Jun N-terminal kinases	Attenuating neovascularization by inhibiting invasion and spreading of HRMECs.	[79]
	Que	Rats (50 mg/kg/day; 5 weeks; OG)	STZ-induced DR rat model	Brain-derived neurotrophic factor, nerve growth factor, AKT, Bcl-2, cytochrome C, and caspase3	Increasing neurotrophic factor levels and inhibiting neuronal apoptosis to protect neurons in the DR.	[137]
	Nano-formulation of Que	Zebrafish (5, 10 mg/kg; 21 days; IP)	STZ-induced DR zebrafish model	GSH	Improvement of DR and neurosensory deficits through antihyperglycemia, modulation of homocysteine pathway, scavenging of free radicals, and reduction of lipid peroxidation.	[138]
	Que	HRMECs (20,40,80 µL; 48 h)	High-glucose-treated HRMEC mimics angiogenesis	NLRP3, Caspase1, LC3, Beclin 1, IL-1β, IL-18	Suppression of DR RNV through inhibition of NLRP3 inflammasome and autophagy signaling pathways.	[139]
	Que	Rats (150 mg/kg/day; 16 weeks; IP)	STZ-induced DR rat model	IL-1β, IL-18, IL-6, TNF-α, NF-κB	Therapeutic effect on DR by increasing HO-1 expression.	[140]
	Que	Rats (40, 80, 120 mg/kg; 12 weeks; OA)	High-fat diet and streptozotocin-induced retinopathy model	NRF2, TNF-α	Reducing the decrease in outer nuclear layer thickness, the levels of pro-inflammatory factors, and blood glucose, increasing the activities of antioxidant enzymes and the concentration of insulin, and inhibiting intestinal dysbiosis.	[141]

Abbreviations: AMD, age-related macular degeneration; RP, retinitis pigmentosa; DR, diabetic retinopathy; IP, intraperitoneal injection; IVI, intravitreally injection; IG, intragastric administration; OA, oral administration; OG, oral gavage; IV, intravenous administration; NaIO3, sodium iodate; NRF2, nuclear factor erythroid 2-related factor 2; PGC-1α, peroxisome proliferator-activated receptor γ coactivator 1-alpha; SIRT1, silent information regulator 1; HO-1, heme oxygenase-1; NQO1, NAD(P)H quinone oxidoreductase-1; GCL, glutamate cysteine ligase; Que, quercetin; Que-PC, complex formed by the combination of Que and phospholipids; Que-SD, solid dispersion of Que-PC; ARPE-19 cells, human retinal pigment epithelium cells; IL, interleukin; RPE, retinal pigment epithelial; Bcl-2, B-cell lymphoma 2; Bax, Bcl-2-associated X protein; A2E, N-retinylidene-N-retinylethanolamine; PI3K, phosphatidylinositide 3-kinase; AKT, protein kinase B; ROS, reactive oxygen species; NF-κB, nuclear factor kappa-B; Keap1, Kelch-like ECH-associated protein 1; ARE, antioxidative response element; CNV, choroidal neovascularization; Que-7-ALA, Que-7-α-linolenic acid; Que-3-LA, Que-3-linoleic acid; Que/mR150-NSLNs, asparagine–glycine–arginine peptide-modified solid lipid nanoparticles for co-delivery of microRNA-150 and Que; HUVECs, human umbilical vein endothelial cells; VEGF, vascular endothelial growth factor; D+Que, dasatinib plus Que; LCZ, a mixture of luteine/β-carotene/zinc gluconate; EMT, epithelial–mesenchymal transition; 661W cells, mouse cone photoreceptor cells; GSK-3β, glycogen synthase kinase-3 beta; STAT3, signal transducer and activator of transcription 3; NIH-3T3 cells, mouse embryonic fibroblast cells; RHO, rhodopsin; COS-1 cells, African green monkey SV40 transformed kidney cells; RGCs, retinal ganglion cells; Que@TPP-ROS-Lips, encapsulated Que in ROS-responsive mitochondria-targeted liposomes; FOXO3A, forkhead box O3a; p38 MAPK, protein 38 mitogen-activated protein kinase; RES- and Que-loaded PEG-modified CS NPs, Que loaded in chitosan nanoparticles modified by natural antioxidant resveratrol; 8MQPM, a permethylated form of Que; Q-IP-DHA, 3-O-docosahexaenoicacid-7-O-isopropyl-quercetin; M-Q-IP-DHA, micelles-Q-IP-DHA; LNC-Q-IP-DHA, lipid nanocapsules-Q-IP-DHA; HRMECs, primary human retinal microvascular endothelial cells; ERK, extracellular signal-regulated protein kinase; STZ, streptozotocin; TNF-α, tumor necrosis factor α; GSH, glutathione; NQ, nano-formulation of Que; NLRP3, NLR thermal protein domain-associated protein 3.

## Data Availability

Not applicable.
